# Reunion of Cut off Tongue

**Published:** 1890-10

**Authors:** 


### REUNION OF CUT OFF TONGUE.

Dr. N. C. Davis, of Good Thunder, Minn., in September, 1884, was summoned to see a boy seven years of age who had been kicked by a horse on the right cheek, breaking off the first bicuspid tooth. The tongue was cut entirely off at the junction of the tip with the base, or the posterior portion of the *frœnum linguœ,* with the exception of a few fibres of the tongue and mucous membrane on the right side. When Dr. Davis arrived, the end of the tongue was protruding from the mouth. The hemorrhage was controlled by a dilute solution of persulphate of iron. He sent for Dr. J. W. Andrews to assist him. In about an hour he arrived and administered chloroform. Dr. Davis drew the base of the tongue forward with a tenaculum. Then the apex was brought into opposition with the base, and secured by five silk ligatures above on the dorsum, and seven below. The boy stood the operation well, and the hemorrhage was trivial. The balance of the treatment consisted in syringing out the mouth twice daily with a solution of boracic acid and putting patient upon a liquid diet. The tongue healed nicely, with the exception of a small portion on the left side, which sloughed out and left a small notch, which was nearly replaced by granulation. The doctor discharged the patient in about three weeks with the tongue full length and articulation good.—*Northwestern Med. Journal.*

